# SleepPos App: An Automated Smartphone Application for Angle Based High Resolution Sleep Position Monitoring and Treatment

**DOI:** 10.3390/s21134531

**Published:** 2021-07-01

**Authors:** Ignasi Ferrer-Lluis, Yolanda Castillo-Escario, Josep Maria Montserrat, Raimon Jané

**Affiliations:** 1 Institute for Bioengineering of Catalonia (IBEC), Barcelona Institute of Science and Technology (BIST), 08028 Barcelona, Spain; ycastillo@ibecbarcelona.eu; 2 Centro de Investigación Biomédica en Red de Bioingeniería, Biomateriales y Nanomedicina (CIBER-BBN), 28029 Madrid, Spain; 3 Department of Automatic Control (ESAII), Universitat Politècnica de Catalunya-Barcelona Tech (UPC), 08028 Barcelona, Spain; 4 Sleep Lab, Pneumology Service, Hospital Clínic de Barcelona, 08036 Barcelona, Spain; jmmontserrat@ub.edu; 5 Centro de Investigación Biomédica en Red de Enfermedades Respiratorias (CIBERES), 28029 Madrid, Spain

**Keywords:** accelerometry, biomedical signal processing, mHealth, monitoring, sleep position, smartphone, app, android

## Abstract

Poor sleep quality or disturbed sleep is associated with multiple health conditions. Sleep position affects the severity and occurrence of these complications, and positional therapy is one of the less invasive treatments to deal with them. Sleep positions can be self-reported, which is unreliable, or determined by using specific devices, such as polysomnography, polygraphy or cameras, that can be expensive and difficult to employ at home. The aim of this study is to determine how smartphones could be used to monitor and treat sleep position at home. We divided our research into three tasks: (1) develop an Android smartphone application (‘SleepPos’ app) which monitors angle-based high-resolution sleep position and allows to simultaneously apply positional treatment; (2) test the smartphone application at home coupled with a pulse oximeter; and (3) explore the potential of this tool to detect the positional occurrence of desaturation events. The results show how the ‘SleepPos’ app successfully determined the sleep position and revealed positional patterns of occurrence of desaturation events. The ‘SleepPos’ app also succeeded in applying positional therapy and preventing the subjects from sleeping in the supine sleep position. This study demonstrates how smartphones are capable of reliably monitoring high-resolution sleep position and provide useful clinical information about the positional occurrence of desaturation events.

## 1. Introduction

Sleep is a very important state for living organisms. It occupies approximately one third of our life [1,2,3] and it is known to affect emotional, physical and cognitive performance [4]. There have been many studies highlighting that poor sleep quality or disturbed sleep is associated with multiple health complications. These complications range from mental disorders [5,6,7] to cardiovascular and cerebrovascular diseases [8,9,10], which are the leading cause of death worldwide [11,12].

Sleep position is an important factor affecting the severity and occurrence of multiple diseases, which impacts sleep quality. It has been reported how lateral and prone sleep positions influence the occurrence of dry eye, ocular hypertension, and glaucoma [13,14,15]. Additionally, in late pregnancy, supine sleep position can lead to a reduced flow to the placenta [16,17] and result in poor fetal outcome, affecting fetal growth and increasing the risk of stillbirth, pre-eclampsia/eclampsia, gestational diabetes mellitus, cardiomyopathies and heart failure [18,19]. Furthermore, supine sleep position is also known to promote the occurrence of apneas when compared to lateral and prone sleep positions, in a phenomenon known as positional obstructive sleep apnea (pOSA) [20,21]. Obstructive sleep apnea (OSA) is one of the most common diseases affecting sleep quality and is characterized by repetitive episodes of total or partial air pathway obstruction, which reduce the airflow and lead to hypoxia [22,23,24,25].

Multiple techniques can be used to monitor sleep quality and sleep position. There exist many questionnaires, such as the STOP-Bang [26] and Berlin [27] questionnaires, among others [28], which aim to determine the state of the patient and quantify the sleep quality. These questionnaires can include questions about sleep position, but they fail to properly determine the different sleep positions during the night and are not reliable enough due to their subjective nature [29,30,31]. Other techniques, such as polysomnography (PSG), which is the gold-standard test for diagnosing sleep-related diseases, use accelerometry-based sensors, as well as cameras, for monitoring sleep position, which leads to a more objective and reliable measure. However, the position obtained from PSG usually requires the validation of sleep technicians to properly determine the sleep position. Moreover, the PSG test is known to promote supine sleep position [32,33], due to the amount of wires and devices used. There are also studies which aimed to use a combination of video cameras and infrared cameras to reliably monitor and detect the different sleep positions in an unobtrusive manner [34,35,36]. However, these approaches require specific cameras to be installed on the bedframe or in the room.

Even though there exist multiple techniques to monitor sleep position, it is usually only classified into four discrete categories: supine, prone, lateral left, and lateral right. Recent studies have demonstrated that a higher resolution sleep position, provided with an angular resolution, would be necessary to better diagnose pOSA [37,38] as there could exist variability within the four clinically used sleep positions categories (supine, prone, left and right).

Positional treatment is a procedure by which a patient is forced to avoid sleeping in a certain sleep position. It is one of the less invasive methods to treat multiple diseases [39,40,41], and has proven to be efficient in patients suffering pOSA [42,43]. There are multiple ways to achieve it, including the use of bands covering the thorax which contain objects inside them to prevent certain specific sleep positions, such as the tennis ball technique (TBT) for pOSA [44,45]. Other approaches attach devices to the body which vibrate when sleeping in a certain position [46,47,48] to force the patient to change it. These devices achieved the same results as the TBT, but with improved sleep quality, quality of life, and compliance [49], as the long-term adherence of the TBT is poor [50].

Smartphones have appeared in the last years as tools with a great potential to monitor healthy and disturbed sleep [51,52]. They have great advantages, such as containing a lot of embedded sensors, and being worldwide available. Multiple approaches have already been made, including some previous work of our group, in developing tools to monitor sleep quality. These attempts included the analysis of audio to detect snoring and breathing [53,54,55,56], the analysis of accelerometry to detect chest movement, respiration and sleep depth [57,58,59,60], and, sometimes, the combination of both with portable oximetry devices. Furthermore, smartphones have been tested as vibrational position treatment devices [61]. Nevertheless, despite the multiple approaches using smartphones to determine sleep quality, none have focused in providing high-resolution reliable sleep position and positional treatment at the same time.

The aim of this research is to develop a smartphone application that can be used to reliably monitor sleep position and detect the positional occurrence of desaturation events and perform positional treatment. We divided our study into three tasks: (1) develop an Android smartphone application (‘SleepPos’ app) which monitors angle-based high-resolution sleep position and provides the option to simultaneously apply positional treatment; (2) test the smartphone application at home coupled with a pulse oximeter; and (3) explore the potential of this tool when combined with a pulse oximeter to detect the positional occurrence of desaturation events.

## 2. Materials and Methods

### 2.1. ‘SleepPos’ App: Design and Specifications

We developed ‘SleepPos app’, an Android application to monitor high-resolution sleep position and eventually provide positional treatment to the patients.

#### 2.1.1. Hardware

**Figure 1:**
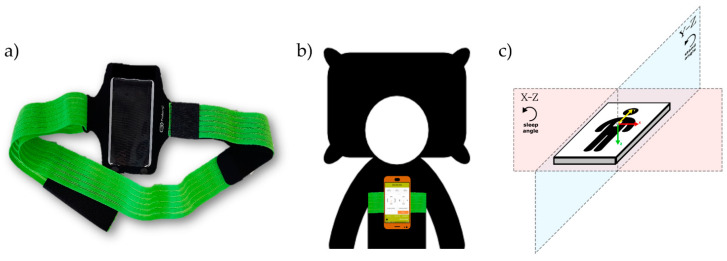
Image of the fixation system (**a**), composed of an elastic band and a bag which allows to place the smartphone over the sternum (**b**) in the desired configuration to run the ‘SleepPos’ app. The smartphone accelerometry sensor should be providing positive values (**c**) when accelerations occur from right to left (X-axis, red arrow), from toe to head (Y-axis, yellow arrow), and from front to back (Z-axis, green arrow), defining the X–Z plane, where the sleep angle is calculated, and the Y–Z plane, where the stand angle is calculated.

Two different elements are needed to use the ‘SleepPos’ app:

1. An Android smartphone containing an accelerometry sensor.
2. A fixation system to place the smartphone over the sternum, in the configuration proposed by Nakano et al. [53] and successfully tested by our group [55,59]. We suggest the use of an elastic band for that purpose, as shown in Figure 1a.

An example of the placement of these different elements can be seen in Figure 1b, where the elastic band proposed is placed over the sternum and contains a smartphone inside with the ‘SleepPos’ app. In Figure 1c we can observe how the accelerometry sensor should be placed for the app to function properly. The smartphone accelerometry sensor should be providing positive values when the acceleration goes from right to left (X-axis, red arrow), from toe to head (Y-axis, yellow arrow), and from front to back (Z-axis, green arrow), thus defining the X–Z plane, where the sleep angle is calculated, and the Y–Z plane, where the stand angle is calculated. The sleep angle provides information about the sleep position and the rotation of the subject while sleeping. With this configuration, a sleep angle of 0° belongs to a pure left sleep position, 90° belongs to a pure supine position, ±180° belongs to a pure right position, and −90° belongs to a pure prone position. The stand angle provides information whether the subject is standing or lying in bed, and, with this configuration, a stand angle of ±180° belongs to a stand position, 0° belongs to a headstand position, and 90° and −90° belong to a pure laying position. The developed app allows visual validation of each sleep position orientation, to ensure that the smartphone is being used correctly, and is explained in the following section.

#### 2.1.2. Software

The software used to develop the Android application was ‘Android Studio’ (Google, Mountain View, United States), a free-to-use Android integrated development environment, under the Apache 2.0 license.

The Android application developed can be installed starting from Android Lollipop version (Android 5.0), which is expected to cover, at least, 94.1% of the Android smartphone devices on the market. This app has been developed with the experience and feedback provided by subjects from previous studies of our own group [38,54,55,58,59].

**Figure 2:**
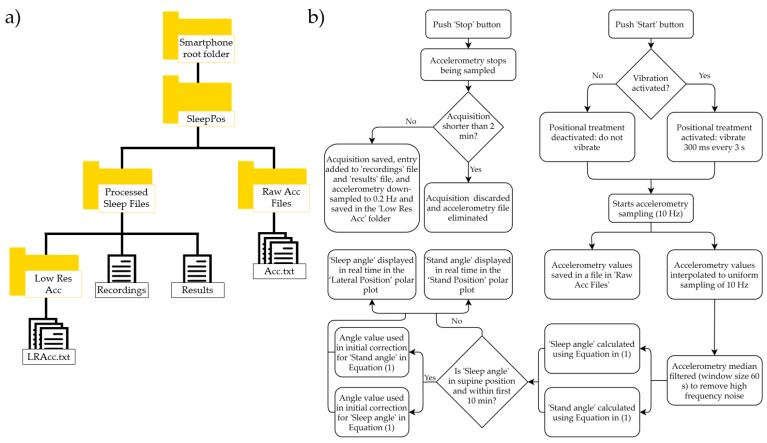
Folder structure of the app (**a**) used to store the raw and processed information from the accelerometry sensor of the smartphone. Raw accelerometry is saved within the ‘Raw Acc Files’ folder. The ‘Recordings’ file contains the register of the multiple recordings performed. The ‘Results’ file contains the register with the summarized processed information from the accelerometry, and the ‘Low Res Acc’ folder contains the low sampling frequency accelerometry used for data visualization. The diagram in (**b**) shows the logic applied in the app to acquire and process the accelerometry data.

This application can run in background mode with the screen switched off. In its first use, the Android application checks that the necessary Android permissions to use the application are granted. It also reminds the user the need to disable battery optimization for this app, to avoid restrictions applied by the Android doze mode and allow the accelerometer sensor to continuously acquire data during the whole night. Then, it generates the folder structure to save the accelerometry data acquired and processed. The folder structure can be seen in Figure 2a. Once these aspects have been fulfilled, the app displays three tabs: ‘Acquisition’, ‘Recordings’, and ‘Results’.

1 ‘Acquisition’ tab: this tab allows to start the sleep position acquisition by clicking the ‘Start’ button and stop it by clicking the ‘Stop’ button. Once the ‘Start’ button is clicked, a dialog prompts asking if you would like the smartphone to vibrate when sleeping in a supine-like sleep position, to force the user to change the sleep position. If vibration is selected, when sleeping in a supine-like sleep position, the smartphone will vibrate for 300 ms every 3 s. After the user’s decision upon the vibration option, accelerometry starts being sampled at a non-uniform sample rate of around 10 Hz. The acquired accelerometry data are saved in a separate file in the ‘Raw Acc Files’ folder seen in Figure 2a, and a new registry is entered in the ‘Recordings’ file. Then, it is simultaneously processed to extract information of interest and it is interpolated to a uniform sampling rate of 10 Hz. Afterwards, each sampled accelerometry value is median filtered, with a window of 60 s around each sample, to remove the high frequency noise from the accelerometry. In addition, the sleep angle and stand angles are calculated and displayed on the polar plots from the tab using the following formula:
  (1) $$\mathrm{Angle}\;(º)=\frac{180}{\Pi }\cdot \mathrm{acos}\left(\frac{\vec{a}\cdot \vec{b}}{\left|\vec{a}\right|\cdot \left|\vec{b}\right|}\right)\cdot sign(c)+\theta_{Initial}$$
  where, for the sleep angle, $\vec{a}=\left(X_{n},Z_{n}\right)$ with $X_{n}$ and $Z_{n}$ being the values of the X and Z axis of the triaxial accelerometry at each specific timestamp; $\vec{b}=\left(1,\;0\right)$, which is a static reference aligned with the left sleep position; and $c=Z_{n}$, to be able to differentiate supine and prone positions. For the stand angle, $\vec{a}=\left(Y_{n},Z_{n}\right)$, with $Y_{n}$ and $Z_{n}$ being the values of the Y and Z axis of the triaxial accelerometry at each specific timestamp; $\vec{b}=\left(1,\;0\right)$, which is a static reference aligned with the stand position; and $c=Z_{n}$, to be able to differentiate stand and headstand positions. The $\theta_{Initial}$ is an angle used to correct the differences in anatomy between patients when placing the phone over the sternum. It is automatically determined from the sleep and stand angles when sleeping in a supine position during the first 10 min of acquisition [38]. It can be different for the sleep and stand angles and it is used to ensure that the smartphone returns 90° for both the sleep and stand angles when being in a pure supine position, which are the values expected for this position. The algorithm described in this section has successfully been tested and validated in a previous work of our group compared to the video-validated position from the PSG [38].
   Then, the sleep angle is used to display the high-resolution sleep position in real time in the ‘Sleep Position’ polar plot, which shows the smartphone angular orientation between the four classic sleep positions (supine, left, right and prone). The stand angle can also be observed in real time to determine if the subject is laying on the bed or standing, by looking at the ‘Stand Position’ polar plot, which shows the angular orientation between the standing and laying positions. The acceleration values and time spent on the acquisition can also be seen in this Table Finally, accelerometry is down-sampled to 0.2 Hz and saved also in the ‘Low Res Acc’ folder for its visualization in the results Table Acquisitions shorter than 2 min are discarded. The logical scheme applied in this tab can be seen in Figure 2b.
2 ‘Recordings’ tab: this tab displays all the sleep position acquisitions performed. For each acquisition, the date, start time, and duration are shown. It is also possible to switch to a calendar view, where the number of acquisitions per day are shown. Finally, in this menu it is possible to observe whether the accelerometry file is still saved in the smartphone memory and it gives you the option to delete the file and/or the results linked to the acquisition.
3 ‘Results’ tab: this tab shows the sleep position results for each of the acquisitions, including the acquisition date, start time, and a summary of the discrete sleep position performance. When clicking on each of the sleep position summary boxes, a dialog box appears showing the details of the acquisition. The details of the sleep position include a polar plot showing the evolution in time of the sleep position as well as a summary table with the minutes and percentage of minutes spent at each sleep position. In the detailed polar plot, the standing position, if present, appears in blue, and the laying position in orange. The high-resolution sleep position orientation is also shown, allowing to determine the exact angular orientation at each specific time of the night.

### 2.2. ‘SleepPos’ App: Position Monitoring during the Night

The acquisition protocol used for all the experiments in this study was approved by the ethics committee from the Hospital Clínic of Barcelona. We have tested the ‘SleepPos’ Android app in 17 subjects who volunteered to use it during a whole night in sleep position diagnosis mode. These volunteers were proposed by the Hospital Clínic of Barcelona, and some of them were suspected of having positional OSA. The database used for the analysis is composed of 11 men and 6 women, with an average age of 51 (24–83) and an average BMI of 27.7 (20–34). They were given a Samsung S5 SM-G900F Android 6.0.1 smartphone (Samsung, Seoul, South Korea) with the app installed on it, a wired pulse oximeter Nonin 8000s (Nonin, Plymouth, MN, USA), and the fixation system proposed shown in Figure 1a.

From the app we obtained the angular-based high resolution sleep position, the percentage of time and minutes spent lying and standing, and the percentage of time and minutes spent for each discrete sleep position (supine, prone, left and right). We also processed the raw accelerometry files saved within the ‘Raw Acc Files’ folder (Figure 2a) with the MATLAB r2019b software (Mathworks Inc., Natick, MA, USA). We developed custom algorithms to calculate the mean angle ($\bar{X}$) and standard deviation ($\sigma_{X}$) of each of the sleep angles within each discrete sleep position (supine, prone, left and right) to study the angular variation at each discrete sleep position and determine whether they were steady at a specific position or not.

### 2.3. ‘SleepPos’ App: Combination with Oximetry

We used the acquisition protocol explained in the previous subsection to study the occurrence of desaturation events linked to the angular-based high resolution sleep position. The desaturation events are defined as the time intervals that the blood oxygen level has a drop of at least 3%, respect to the baseline prior to this drop, in a short period of time (maximum 120 s) due to disordered breathing [62,63]. To perform this study, we also developed custom-made algorithms with MATLAB r2019b. For each of the 17 subjects, we obtained the desaturation events provided by the pulse oximeter and calculated the percentage of time spent in a certain sleep position angle with the following formula:

(2) $${\%\mathrm{Position}}_{\theta }=100\cdot \;\;\frac{\sum_{i=1}^{N}{\mathrm{Position}}_{i}[{\mathrm{Angle}}_{\theta }^{low}\leq {\mathrm{Position}}_{i}\leq {\mathrm{Angle}}_{\theta }^{high}]}{N}\;$$

where ${\%\mathrm{Position}}_{\theta }$ provides the percentage of time spent in a certain sleep angle *θ*; *N* is the number of sleep position samples available; ${\mathrm{Position}}_{i}$ represents each sleep position sample matching the criteria between brackets; and the ${\mathrm{Angle}}_{\theta }^{low}$ and ${\mathrm{Angle}}_{\theta }^{high}$ are the thresholds used around each sleep angle *θ*, forming a window of 15° (*θ* ± 7.5°). The evaluated sleep angles *θ* ranged from −180° to 180° with 1° increase.

We also calculated, for each patient, the percentage of desaturation events (DE) at a certain sleep position angle. To achieve this, we took each desaturation event and assigned it the mean angle value of all the sleep position angle samples that occurred during the event. Afterwards, we calculated the percentage of events occurring at a certain angle with the following Equation:

(3) $${\%\mathrm{DE}}_{\theta }=100\cdot \;\;\frac{\sum_{i=1}^{N}{\mathrm{DE}}_{i}[{\mathrm{Angle}}_{\theta }^{low}\leq {\mathrm{DE}}_{i}\leq {\mathrm{Angle}}_{\theta }^{high}]}{N}\;$$

where ${\%\mathrm{DE}}_{\theta }$ is the percentage of events in a certain sleep angle *θ*; *N* is the number of desaturation events available; ${\mathrm{DE}}_{i}$ represents the median angle linked to an event matching the criteria between brackets; and the ${\mathrm{Angle}}_{\theta }^{low}$ and ${\mathrm{Angle}}_{\theta }^{high}$ are the thresholds used around each sleep angle *θ* with the same window and resolution as Equation (2).

Finally, to determine the relationship between the occurrence of the desaturation events and the sleep position, two variables were calculated linked to their angle of occurrence: the local oxygen desaturation index (ODI) and the ratio between the percentage of events and the percentage of position. The formulas used to calculate these two variables were the following:

(4) $${\mathrm{Local}\;\mathrm{ODI}}_{\theta }=\;\frac{\sum_{i=1}^{N}{\mathrm{DE}}_{i}[{\mathrm{Angle}}_{\theta }^{low}\leq {\mathrm{DE}}_{i}\leq {\mathrm{Angle}}_{\theta }^{high}]}{\frac{1}{36000}\cdot \sum_{j=1}^{M}{\mathrm{Position}}_{j}[{\mathrm{Angle}}_{\theta }^{low}\leq {\mathrm{Position}}_{j}\leq {\mathrm{Angle}}_{\theta }^{high}]}\;$$

(5) $${\mathrm{Ratio-}\%\mathrm{DE}/\%\mathrm{Position}}_{\theta }=\;\frac{{\%\mathrm{DE}}_{\theta }}{{\%\mathrm{Position}}_{\theta }}\;$$

where the ${\mathrm{Local}\;\mathrm{ODI}}_{\theta }$ provides an estimation of the ODI in a certain sleep angle *θ*; *N* is the number of desaturation events available; ${\mathrm{DE}}_{i}$ represents the median angle linked to an event matching the criteria between brackets; ${\mathrm{Angle}}_{\theta }^{low}$ and ${\mathrm{Angle}}_{\theta }^{high}$ are the thresholds used around this angle with the same window and resolution as Equation (2); the coefficient $\frac{1}{36,000}$ is used to normalize the position to hours, taking into account the sampling frequency of the smartphone (10 Hz); *M* is the number of sleep position samples available; and ${\mathrm{Position}}_{i}$ represents each sleep position sample matching the criteria between brackets. The ${\mathrm{Ratio-}\%\mathrm{DE}/\%\mathrm{Position}}_{\theta }$ provides information about the occurrence of events at a certain sleep angle *θ*; and ${\%\mathrm{DE}}_{\theta }$ and ${\%\mathrm{Position}}_{\theta }$ are the results from Equations (2) and (3) for each angle *θ*. To avoid high-value artifacts due to denominators being close to 0 in both the ${\mathrm{Local}\;\mathrm{ODI}}_{\theta }$ and ${\mathrm{Ratio-}\%\mathrm{DE}/\%\mathrm{Position}}_{\theta }$ variables, a minimum denominator value is used in both equations, which is 10 min for the ${\mathrm{Local}\;\mathrm{ODI}}_{\theta }$ and 1% for the ${\mathrm{Ratio-}\%\mathrm{DE}/\%\mathrm{Position}}_{\theta }$. Moreover, the ${\mathrm{Local}\;\mathrm{ODI}}_{\theta }$ is smoothed with the mean value calculated from the window containing the previous and following two values.

### 2.4. ‘SleepPos’ App: Positional Treatment

To validate the efficacy of the vibration as a positional treatment strategy to avoid sleeping in the supine sleep position, nine subjects were tested some months later with the vibration option activated and compared to their previous sleep position recording without vibration. The vibration characteristics were described in the Section 2.1.2 “‘Acquisition’ tab” of this study. We used the same acquisition protocol explained for the previous subsection, with an EMO-80 wireless pulse oximeter (EMAY Ltd., Hong Kong, China), and determined the high-resolution sleep position and the desaturation events of at least 3%. To perform this study, we also developed custom-made algorithms with MATLAB r2019b.

We have used the Equations (2)–(5) to determine the effect of the positional treatment in the appearance of desaturation events and to assess the ODI values of each subject linked to their sleep position angle.

## 3. Results

### 3.1. ‘SleepPos’ App: Design and Specifications

**Figure 3:**
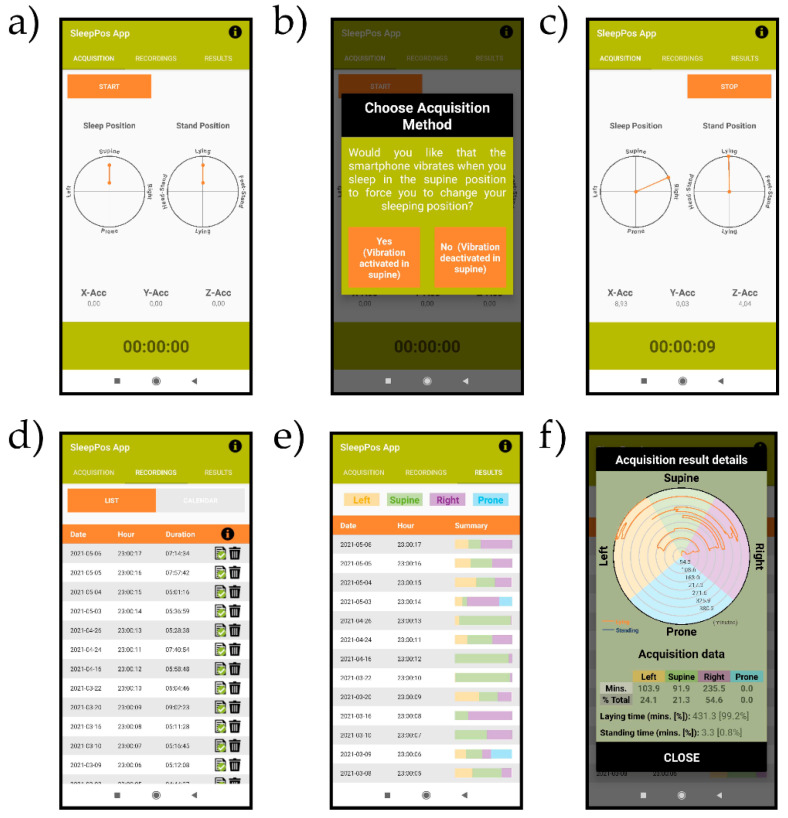
Screenshots of the ‘SleepPos’ app. The ‘Acquisition’ tab (**a**) is used to record the sleep position. Upon clicking the ‘Start’ button, a dialog is prompted (**b**) asking if positional treatment should be applied. Afterwards, the sleep position is acquired and displayed (**c**) on the polar plots as well as the accelerometry values and the time spent on the acquisition. The ‘Recordings’ tab (**d**) shows the details of each acquisition, which include the date, hour of start, and duration. The ‘Results’ tab (**e**) shows a summary of the sleep position for each acquisition. When clicking over each summary, a window prompts (**f**) showing the sleep position with angular resolution, as well as a table with the summarized details of the acquisition.

Screenshots of the ‘SleepPos’ app are shown in Figure 3. The ‘Acquisition’ tab (Figure 3a) allows to start the acquisition of the sleep position. Once the ‘Start’ button is clicked, a window prompts (Figure 3b) asking if positional treatment should be applied. If the user selects positional treatment, the smartphone will vibrate for 300 ms every 3 s when sleeping in a supine like position, to induce the user to change the sleep position. An example of a real time representation of the sleep position and whether the subject is standing or not can be seen in Figure 3c. This allows to check if the smartphone has been placed correctly to monitor the sleep position during the night.

The ‘Recordings’ tab (Figure 3d) shows the list of recordings performed with their acquisition date, hour of start and duration. The icon 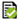 is used to provide the information that the raw accelerometry is still saved in the ‘Raw Acc Files’ folder, whereas the 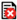 icon is used to provide the information that the raw accelerometry has been removed. The icon 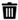 opens a window which let the users decide whether to remove the raw accelerometry file to reduce the smartphone’s memory used, or to remove this acquisition and its results from the app. Each acquisition is estimated to occupy at maximum 20 Mb.

Finally, the ‘Results’ tab (Figure 3e) shows the outcomes of the sleep position performance for each acquisition. To easily identify the results the user wants to check, the date and hour of start are provided for each acquisition. When the user clicks over any of the summary boxes displayed for each acquisition, a window appears (Figure 3f) showing the sleep position with angular resolution, as well as a summary table with the minutes and percentage of time spent in each sleep position. It also displays the minutes and percentage of time spent lying or standing.

### 3.2. ‘SleepPos’ App: Position Monitoring during the Night

**Figure 4:**
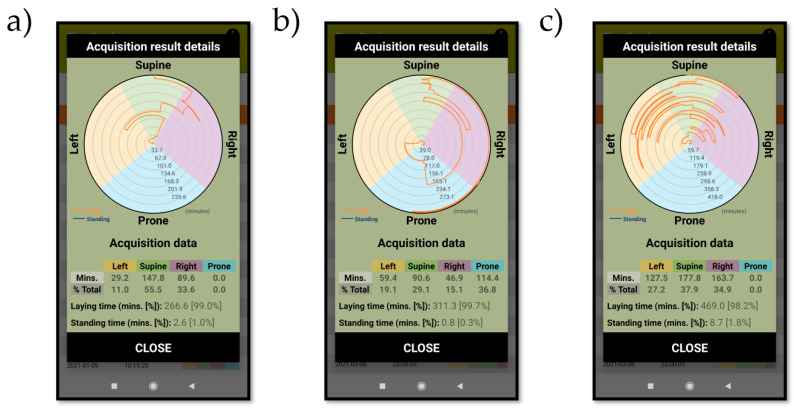
Screenshots of the ‘SleepPos’ app results for subject 2 (**a**), 6 (**b**) and 16 (**c**). For each subject, the angular sleep position is provided, as well as the summary of results for each sleep position. It is possible to observe how subject 2 (**a**) and 6 (**b**) have few position changes, whereas the subject 16 (**c**) has a lot of position changes. This behavior is only noticeable on the angular-based high-resolution sleep position as it is not possible to be seen in the summary table.

Figure 4 shows the sleep position results for subjects 2 (Figure 4a), 6 (Figure 4b) and 16 (Figure 4c). For each subject, we can observe the angular-based high resolution sleep position, as well as the summary of the discrete sleep position performance. We can see how subject 2 was lying in bed for 266.6 min (~4 h 30 m) and spent 11% of this time in a left sleep position, 55.5% in a supine sleep position and 33.6% in a right sleep position. Subject 6 spent 311.3 min (~5 h 10 m) lying in bed and spent 19.1% of this time in a left sleep position, 29.1 in a supine sleep position, 15.1% in a right sleep position and 36.8% in a prone sleep position. Finally, subject 16 spent 469.0 min (~7 h 45 m) lying in bed and spent 27.2% of this time in a left sleep position, 37.9% in a supine sleep position and 34.9% in a right sleep position. It is noticeable how subject 2 spent most of the time (>50%) in a supine sleep position compared to subjects 6 and 16. Additionally, subject 6 spent 36.8% of the time (~2 h) sleeping in a prone sleep position, which was its preferred sleep position compared to the time spent in the lateral positions (106.3 min, 34.2% of the time) or in the supine position (90.6 min, 29.1% of the time). Finally, the angular-based high-resolution sleep position reveals how subject 16 has a lot of position shifts when compared to subjects 2 and 6. It also reveals the existence of angular differences within the supine sleep position and within the right sleep position in subject 2. These angular differences within the same sleep position can also be seen in subject 6 and 16. This indicates how a high-resolution sleep position would be needed to better determine the sleep position and the movement of the subject when sleeping.

Table 1 shows the summary of the discrete sleep position performance for all the subjects included in this study. The minutes and percentage of time spent lying or standing, and at each of the sleep positions were obtained from the ‘SleepPos’ app. From the raw accelerometry files provided by the ‘SleepPos’ app, we also calculated the mean and standard deviation of the angles obtained for each sleep position and patient. When observing the table, we can determine that the supine sleep position was only the preferred option for 9 of the 17 subjects, and only 3 of these subjects (10, 12 and 13) spent more than 60% of the time at that specific sleep position. It is also noticeable how only 3 subjects (1, 6 and 14) slept occasionally in a prone-like sleep position. Subjects 6 and 14 spent a reasonable amount of time in the prone position, but subject 1 spent only 2.3 min, which is likely that the time spent on that sleep position was part of a transition between right and left sleep positions through the prone sleep position.

When looking at the mean and standard deviation values for the angles within each sleep position, we can observe how the supine position has less variability when compared to the lateral sleep positions. It is also noticeable how the lateral sleep positions have a wider range of values for the mean angle (−22.1° to 59.2°; and −173.1° to 125.5°) when compared to the supine sleep position (85.2° to 112.4°). This indicates that, while the supine position is more similar between all the patients on the database, the lateral positions are more different.

Finally, we can also observe how the duration of the ‘SleepPos’ app sleep positions assessed in this study ranged from the lowest value of 4 h 29 m in subject 2, to slightly over 9 h in subject 9, with an average time of almost 6 h per test, which is a reasonable amount of time for studying sleep position.

### 3.3. ‘SleepPos’ App: Combination with Pulse Oximetry

In Table 2 we can see that 1967 desaturation events were recorded by the pulse oximeter from all subjects in this study. The subjects with less desaturation events were subjects 5 and 6, with four and six events, respectively. They also had the lower ODI values, 0.8 and 1.2, respectively. Contrarily, the subjects with more desaturation events were subjects 11 and 12, with 295 and 342. They also had the higher ODI values with 39.2 and 57.7, respectively.

If we use the apnea hypopnea index (AHI) severity classification for OSA patients defined by the American Academy of Sleep Medicine (AASM) [62] applied to the ODI values [64], there would be four healthy subjects (5, 6, 9 and 10), three mild subjects (1, 3 and 14), eight moderate subjects (2, 4, 7, 8, 13, 15, 16 and 17) and two severe subjects (11 and 12). This situation would indicate that the database is balanced with at least two subjects in each category. We can also observe that there are multiple subjects close to the thresholds defined by the AASM. For instance, subject 1 is 0.1 below the threshold from being classified from mild to moderate, subject 9 is 0.3 below the threshold from being classified from healthy to mild, subject 13 is 0.3 over the threshold from being classified from moderate to mild, subject 15 is 0.5 over the threshold from being classified from moderate to mild and subject 16 is 0.6 from being classified from moderate to severe.

**Figure 5:**
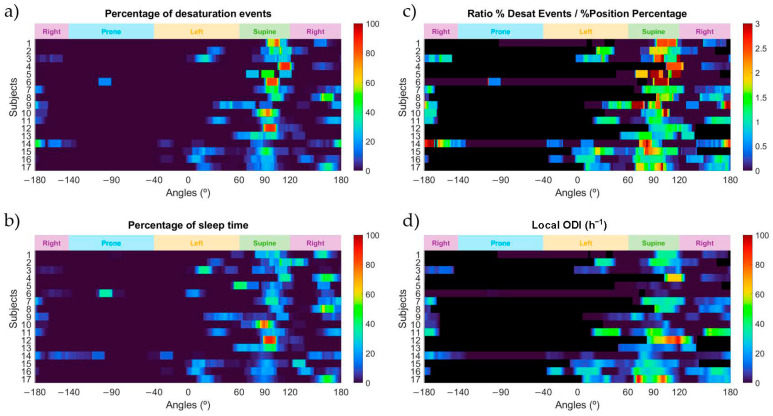
Heatmaps of the percentage of desaturation events (**a**); the percentage of sleep position time (**b**); the ratio between the percentage of desaturation events and the percentage of sleep position time (**c**); and the local ODI (**d**) for each sleep position angle and for each of the subjects in this study. Black colors shown in the heatmap indicate that the ratio and the local ODI could not be calculated for that specific angle due to no time slept on it.

In Figure 5 we can observe the relationship between the appearance of desaturation events and the high-resolution sleep position obtained by the ‘SleepPos’ app for all subjects of the database. Figure 5a shows the percentage of desaturation events appearing at each sleep angle. It is possible to see how subjects 1, 4, 6, 10 and 12 had most of their events in a supine-like position. On the contrary, subjects 8, 14 and 17 had most of their events in lateral right sleep positions. There exist other subjects, such as number 16, with a more spread pattern of apparition of events. In Figure 5b we can observe the distribution of the sleep position for each subject. Subjects 10 and 12 spent most of their time in supine sleep position. Subject 13 also spent most of the time in supine position, but slightly changed the angular orientation and scored multiple different angles within the supine sleep position. Regarding the other subjects, most of them slept in the lateral position. However, it is important to remark that subjects 6 and 14 slept a reasonable amount of time in the prone position.

In Figure 5c we can observe the ratio between the percentage of events and the percentage of sleep position time as a function of angle. This variable allows us to determine the sleep position angles where the occurrence of desaturation events is higher than the percentage of time spent in that sleep position. There are multiple subjects with values over two, indicating that the number of events in that specific sleep position is, at least, doubled. Subjects 5 and 6 have very high ratios linked to their supine sleep position. However, it is likely that these values are affected by these subjects having very few events (4 and 6 respectively). A similar reasoning applies to subject 9, but it scored 42 events in total, which could indicate that has a positional behavior. A clearer positional behavior can be seen in subjects 1 and 4. They have ratios over 2 in a supine-like sleep position and scored 92 and 136 desaturation events, respectively.

To complement this information, it is also relevant to analyze Figure 5d, where the local ODI value is provided. We can observe that the previously mentioned subjects 5 and 6 have a very low ODI, indicating that they are healthy subjects and do not suffer sleep-disordered breathing. In addition, we can see how subject 9 might have some positional appearance of events in the supine position that are close to the threshold in the left position. It is remarkable to notice that subject 4 has a very severe and positional pattern of appearance of desaturation events, with an ODI value of around 70 for the supine position. The same behavior applies for subject 17. In addition, there are other subjects with remarkable ODI indexes in the supine position but with no data to determine if it is positional or not. For instance, for subject 12 it is not possible to determine if the ODI value of around 90 in the supine position might be due to a positional behavior, as there is no time spent in any other position than supine. Finally, there are other subjects without a positional behavior, such as 11 and 16, that the apparition of desaturation events is not linked to the sleep position.

### 3.4. ‘SleepPos’ App: Positional Treatment

In Table 3 we can see the summary of the discrete sleep position for the nine subjects who underwent positional treatment to avoid the supine sleep position in this study. The amount of time in the supine position was reduced for all of them. Subject 1 was the one with most time in the supine position, with 29.7 min representing 6.2% of the time, whereas subject 7 was the one with less time, with less than a minute. In addition, most of the subjects spent less than 10 min in the supine position, which indicates that the positional treatment worked. This can also be seen upon the general values of the database in Table 1 and Table 3. Prior to the application of positional treatment, the subjects slept around 45.6% of the time in the supine position, and after positional treatment, only a 2% of the time, which demonstrates the efficacy of the positional treatment in avoiding the supine sleep position.

In the same table we can see that 691 desaturation events were recorded by the pulse oximeter. The subjects with less events were 5 and 6, with 17 and 18 events, respectively. The subject with more events was subject 2, with 246 desaturation events. Regarding the severity in terms of the ODI values, there are two healthy subjects (5 and 6), five mild (1, 3, 4, 7 and 10), one moderate (13) and one severe (2). Two subjects (2 and 10) changed their severity classification for worse, and two of them (4 and 7) changed it for better. The ODI value also changed slightly for all the other subjects, but without changing the severity classification.

**Figure 6:**
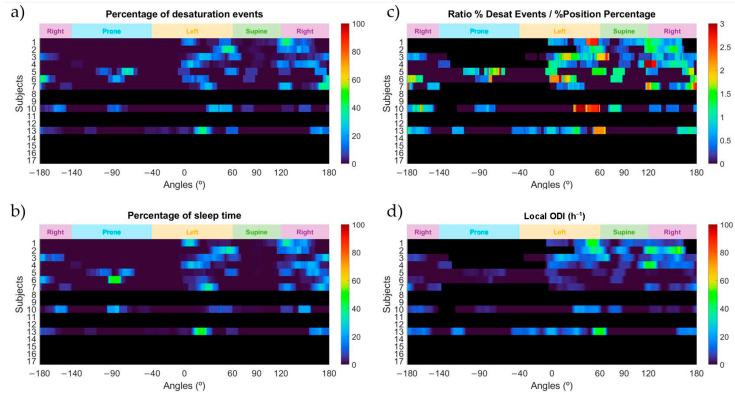
Heatmaps of the percentage of desaturation events (**a**); the percentage of sleep position time (**b**); the ratio between the percentage of desaturation events and the percentage of sleep position time (**c**); and the local ODI (**d**) for each sleep position angle and for the subjects who underwent positional treatment. Black colors shown in the heatmap indicate that either the subject was not treated with the ‘SleepPos’ app positional treatment or the ratio and the local ODI could not be calculated for that specific angle due to no time slept on it.

In Figure 6a we can observe that very few or no events occurred in the supine sleep position. This is related to the information in Figure 6b, where the percentage of time slept at a specific sleep angle is shown. Most of the subjects slept almost no time in the supine sleep position, which demonstrates that the positional therapy was successful in avoiding this sleep position. In Figure 6c we can observe that most of the subjects who underwent positional treatment have values below 2 for the supine sleep position. Subject 4 appears to have values around 3 in the sleep angle values linked to the threshold used to separate the supine and the right sleep positions. This behavior is similar to the one already presented by this subject before applying positional treatment and indicates that a positional behavior is still present. Subject 3 also showed a ratio value over 2 in the sleep position angles around the edge between the supine and left sleep positions and subjects 5 and 6 appear to have values around 1.5, but this is due to the low number of desaturation events they had scored. It is important to consider that the higher ratio values for subjects 1, 3, 4, 10 and 13 appeared very close to the edge between the lateral and supine sleep positions and might indicate that a positional behavior is still present. To complement this information, it is also relevant to analyze Figure 6d, where the local ODI value is provided. Subjects 1, 2, 4 and 13 still seem to present a positional behavior with severe ODI values in the lateral sleep positions close to the supine position, but all the other subjects reveal a healthy or mild behavior. It is important to check how the local ODI values have changed for subject 7 compared to the local ODI values seen in Figure 5d. This subject, when applying positional therapy, clearly reduces the occurrence of desaturation events and reduces its local ODI severity. A similar reasoning applies for subject 4, who after avoiding the supine sleep position, has changed the local ODI from around 70 to around 40 in the sleep angle close to the edge between the supine and left sleep positions. In addition, this allowed reduction of the overall ODI value of the night to less than half of what it was scored before positional treatment (from 25.7 to 12.6).

## 4. Discussion

### 4.1. High-Resolution Sleep Position Monitoring and Treatment Relevance

In our study, we have designed and proposed the use of the ‘SleepPos’ app as a robust method for diagnosing and treating sleep position related diseases. As indicated in previous studies, self-reported sleep position fail to properly determine the sleep position [29,30,31], which is something understandable when looking at Figure 4c and checking the amount of position shifts this subject had and that other subjects might have. In addition, it is very likely that the capacity to keep track of the sleep position is lost once fallen asleep, and thus the reliability of self-reported sleep position remains in doubt. Other techniques, including the PSG and home polygraphy, apply more objective methods and require the use of multiple cameras [34,35,36], which might be expensive and not available to everyone, even though the reliability of these methods is better. Nevertheless, these methods report sleep position classified as one of only four different values: supine, prone, left, and right, and might lack the necessary resolution to properly determine the sleep position [38].

Assuming these limitations, we demonstrated how the ‘SleepPos’ app could provide information regarding the sleep position performance in both a discretized information table and high-resolution angular sleep position graphic. The summarized table information is the method currently used in most clinical departments to assess sleep position. Usually when positional treatment, such as the TBT technique [44,45] or other vibrational devices [46,47,48] are applied to a patient, the efficacy of this technique is validated upon the summarized information on how much time has been spent in a certain sleep position. However, recent studies have demonstrated how a higher resolution sleep position would be needed to better diagnose and monitor the positional occurrence of diseases, such as OSA [37,38], for which we believe that the combination of information provided by the ‘SleepPos’ app allows a better assessment of the sleep position.

We have tested the ‘SleepPos’ app in 17 subjects, and we were able to successfully retrieve their sleep position and assess their sleep performance. Our sample size (17 subjects) does not allow us to extract conclusions which could be extrapolated to the general population as population studies do. However, we consider this limitation to be beyond the scope of this work, as we do not intend to perform a population-based study, but rather a proof-of-concept study on the feasibility of using smartphones to monitor high resolution sleep position with the ability to detect and treat the positional occurrence of desaturation events.

From Table 1 we realized that the prone position was not very common in these patients, as only three subjects had spent time on it, but the overall percentage of time spent in this position in the database (3.2%) was according to the prevalence of this position in population studies [65]. In the same table we can observe how subject 6 and 14 spent 36.8% and 22.7% of their time in the prone sleep position, and in Table 3 we can observe how subjects 5, 6 and 10 spent 37.9%, 48.8% and 29.6% of the time in the prone sleep position, which would indicate that the fixation system proposed in this study should not prevent the subjects from sleeping in the prone sleep position. Another important finding is that only nine subjects spent their majority of time in the supine position. This behavior is different from the one that can be seen at hospitals, as it is known that some of the tests which assess the sleep position, such as PSG, promote to sleep in supine position [32,33]. Regarding the mean angles for each subject and sleep position, it is possible to observe that the lateral sleep positions have a wider range of values for the mean angle (−22.1° to 59.2° and −173.1° to 125.5°) when compared to the supine sleep position (85.2° to 112.4°). This indicates that the supine sleep position is more alike amongst patients than lateral sleep positions. However, we need to consider that the supine sleep position has a smaller angle range than the lateral and prone sleep positions [38]. In addition, the standard deviation values confirm that there exist subjects who move considerably during the night, such as subject 3, 9 and 14. The sleep angle standard deviation ($\sigma_{X}$) values for each of these subjects are quite high compared to other subjects, such as 8, 11, 12 and 17.

Finally, the positional therapy applied by the ‘SleepPos’ app revealed how it was effective in preventing the subjects from sleeping in a supine sleep position. The positional therapy was applied some months later to only nine of the seventeen subjects of the database due to limited availability of the patients. Nevertheless, these nine subjects provided relevant information to determine how the positional treatment worked on them. As there is evidence of the efficacy of vibrational devices in preventing certain sleep positions [46,47,48], the results obtained can be reliably interpretated as a proof-of-concept confirming the feasibility of the app to conduct positional therapy.

Regarding the positional treatment efficacy, it is remarkable to see how subject 10 changed from spending 95.2% of the time in the supine position to only 3.2% of the time when being treated. The same good results were obtained in all the subjects who underwent positional treatment. This demonstrates the efficacy of the vibration in avoiding certain sleep positions, and that the pattern of vibration used was satisfactory in achieving its purpose. All the subjects who underwent positional treatment reported that the vibration pattern used was noticeable, but not annoying, while being awake and in supine position. Once having fallen asleep, none of the subjects reported to be neither awaken nor annoyed by the vibration applied as positional treatment, which is important to avoid poor sleep. In addition, the subjects reported having a comfortable sleep of good quality. Nevertheless, quantitative relation between the sleep quality and the application of positional treatment was not assessed, as no objective measures of sleep quality, such as electroencephalography (EEG), were available.

Nevertheless, knowing that the vibrational devices achieve the same results as other passive techniques, such as the TBT, but with improved sleep quality, quality of life, and compliance [49], the ‘SleepPos’ app proposed in this study could be a feasible solution to be considered as a sleep position trainer.

This reveals the need to monitor sleep positions with a higher resolution, and the necessity to use less obtrusive techniques which allow the monitoring of sleep positions at the usual sleeping environment, which is at home.

### 4.2. Sleep Position and Oximetry

The measurement of oxygen saturation is a widely used technique in medicine nowadays. It has been proved that hypoxia can lead to multiple health complications, increasing the risk to suffer from multiple cerebrovascular and cardiovascular diseases [8,9,10]. There have been multiple studies which revealed how the position can influence the appearance of these hypoxic events [20,21], indicating that position can play an important role. In this study we have explored how the combination of the ‘SleepPos’ app with a medical-grade pulse oximeter could provide relevant information on the number of desaturation events and their occurrence linked to the sleep position.

An important indicator to measure the relevance of the occurrence of desaturation events is the ODI. This index normalizes the number of desaturations by the total amount of time of the acquisition performed and allows to better compare the occurrence of events between subjects. There were four healthy subjects, three mild subjects, eight moderate subjects and two severe subjects in the database according to their ODI value [64], even though multiple subjects had their ODI values close to the threshold values between the four different severity categories. It is important to remark that the ODI could underestimate the severity provided by the AHI, as apneas could occur without the presence of a desaturation event and hypopneas could also be detected linked to an arousal. This situation could significate that the subjects might be more severe in terms of apneas and hypopneas than the ODI severity values reported. Nevertheless, the ODI can usually be used as a good estimator of the sleep apnea severity [64] and can provide clinically relevant information to clinicians. We used the ODI instead of the AHI, as we used a simplified system that only used a pulse oximeter and a smartphone.

Although the ODI is a good metric to determine the severity of the occurrence of desaturation events, it provides a summary of the information of the whole night and does not account for the variability introduced by other factors. For instance, it is known that position plays an important role on the occurrence of apneic events [20,21], which are usually accompanied by hypoxia. In this scenario, the combination of the information on the sleep position obtained from the ‘SleepPos’ app allowed linkage of the occurrence of the desaturation events to its sleep position angle of occurrence. This combination revealed very useful clinical information about the subjects of the database in two different variables: the local ODI and the ratio of the percentage of events versus the percentage of time spent at a specific sleep position angle. These variables allowed us to assess two important aspects: the severity according to the number of desaturations found and the positional behavior. The severity is evaluated by the local ODI and indicates how critical is the situation regarding to the occurrence of hypoxic events, and the positional behavior is assessed by the ratio and indicates if there is a sleep position where these hypoxic events are more prevalent.

When looking at Figure 5, we can observe various subjects with different severities and positional behaviors. Subjects 1 and 4 are cases whose severity varies significantly depending on the sleep position. Both have a severe ODI value at the supine sleep position and a healthy to mild behavior in the lateral sleep positions. It is worth mentioning that subject 4 has an ODI value of around 70 h^−1^ in the supine sleep position, whereas in the right sleep position this value drops significantly to values below 20 h^−1^. This can easily be seen in Figure 5c, where their ratio values are between 2 and 3, indicating that more than double of the desaturation events occurred when compared to the percentage of time spent at that specific sleep position. On the contrary, subject 11 would be a clear case of a severe subject without positional differences. It is possible to observe in Figure 5c how this ratio does not vary between the supine and the lateral sleep positions, and Figure 5d reveals that the ODI is around 40 h^−1^ in all these sleep positions. Subjects 5 and 6 would be a clear case of healthy patients (Figure 5d) with a positional behavior (Figure 5c) since they did around three times more events in the supine sleep position than in the lateral sleep position. However, it is important to notice that these subjects did very few events since they are classified as healthy, and thus the ratio between the percentage of desaturation events and the percentage of sleep position could be overestimated due to this low number of events.

Moreover, there are subjects in which the positional behavior variable cannot be assessed. The reason is that they do not significantly change their sleep position, and it is not possible to detect differences between sleep positions. An example of this case would be subject 12, who sleeps mostly in a supine sleep position, and even though his ODI is very high (around 100 h^−1^), it is not possible to determine if it is increased due to the supine sleep position and if it would be reduced in lateral positions.

After treating some of the subjects of this study with positional treatment provided by the ‘SleepPos’ app, it was reported how the positional occurrence of desaturation events varied. After positional treatment, it is remarkable how subjects 4 and 7 lowered their ODI value from 25.7 to 12.6 and from 26.2 to 6.9. If we compare the behavior of these subjects before and after applying positional treatment we can observe that, by preventing the supine sleep position, most of the events disappeared. Subject 4 still presents a positional behavior around the sleep angles close to the threshold value used between the supine and right sleep positions, as it can be seen in Figure 5c and Figure 6c, but the severity of the local ODI value has varied. When observing the differences in the local ODI values from Figure 5d and Figure 6d we can observe how this subject changed from a value around 70 to a value around 40 in the sleep angle values close to the edge between the supine and right sleep positions. This indicates that this subject could benefit even more of this reduction if the positional treatment therapy were more restrictive regarding which sleep positions should be avoided.

Similar good results can also be seen in subject 1, who also lowered the ODI value. In this case, it is possible to observe that when applying positional treatment multiple events appear close to the supine position, which might indicate that there could still exist some positional behavior, and that a more restrictive positional treatment could lower even more the ODI value. On the other hand, subject 2 appears to have slightly increased the ODI value even after positional therapy. This reveals that this subject might not benefit from positional therapy. Even though this subject increased its ODI value, it is also noticeable that the sleep position of this subject was always close to the supine sleep position, which could also be a reason for the increased appearance of desaturation events. More tests would be needed to better determine if there could exist a positional behavior on this subject. Most of the subjects treated remained with the same severity classification and similar ODI values. These subjects were mostly healthy and mild, and this would indicate that they would not benefit from the positional therapy.

These results suggest that the information obtained by the combination of the ‘SleepPos’ app and the pulse oximeter can provide useful clinical information, and that a high-resolution sleep position is needed to better assess the influence of the sleep position in the appearance of desaturation events.

### 4.3. Smartphones and Their Role as Portable mHealth Tools

Over the last few years, telemedicine has appeared as a promising field for improving the quality of the health system and reducing the associated costs [66]. However, there are multiple technical aspects to be considered and research is still needed in how to better apply it [67]. Derived from telemedicine, mobile health (mHealth) is an interesting solution to provide high quality medical assistance. The use of mobile devices, such as smartphones or tablets, has opened a broad field of study in developing multiple tools for health data acquisitions at home, due to their advantages. Smartphones, which are devices available worldwide, have a lot of sensors which can be used to monitor different health parameters. These sensors can either be embedded or external portable devices, known as wearables.

In our study we developed an Android app, named ‘SleepPos’, which monitors the sleep position with high resolution and allows to perform positional treatment. The monitoring of high-resolution sleep position is important to better determine the occurrence and severity of pOSA [37,38] and could be of high relevance for other diseases. We also studied how this app could be used in combination with a pulse oximeter, to determine the positional occurrence of desaturation events and their severity. Because the availability of portable pulse oximeters is high, the combination of these two devices can be used to assess the sleep quality by means of sleep position and occurrence of sleep events in an easy and affordable manner, providing high relevant clinical information. For our approach, the smartphone has to be placed over the sternum in a configuration already tested in several studies [53,55,59]. To hold the device in position, a fixation system is also needed. We proposed the use of an elastic band, as seen in Figure 1a, which was reported by the subjects who tested it as a comfortable system to hold the smartphone in position while sleeping.

When using smartphones, several aspects should be considered, such as the electromagnetic radiation and the heat that they might produce, as well as the size of the device and their comfort when placed in position. For our study, we configured the smartphones in airplane mode and disabled Wi-Fi and Bluetooth. By adopting these measures, we reduced or removed the risk of harm produced by the possible emissions and heat generated by the smartphone. Regarding the size of the device, the smartphone used measured 142 × 72.5 × 8.1 mm, and neither any issues related to the comfort of the system, nor issues related to problems regarding the radiation or heat emission were reported by any of the subjects of the database. The same positive feedback was obtained regarding the comfort of the pulse oximeters used for this study. It is important to underline that the system proposed in our research is of a smaller size and weight than multiple portable devices for home polygraphy, and we expected the comfort to be, at least, the same. Another important aspect to consider when using smartphones is the battery capacity and the maximum duration of the acquisitions. The smartphones used in this study contained a battery of 2800 mAh, and each acquisition of around 7 h reduced the battery by approximately 30%. This power consumption ensures that the smartphone can run for approximately 24 h straight, which would allow it to perform at least three acquisitions without having to charge the phone.

Finally, the design of the ‘SleepPos’ Android app allows retrieval of high resolution sleep positions, and its use combined with a pulse oximeter can provide relevant clinical information for pOSA detection. To sum up, the novel results presented in this study reinforce the idea that smartphones are promising mHealth tools to be used at home providing relevant information for sleep medicine.

## 5. Conclusions

In our study we developed an Android application, named ‘SleepPos’, which allows to monitor high-resolution sleep position and apply positional treatment by vibration. We combined this app with a pulse oximeter, and we were able to determine the occurrence of desaturation events linked to the sleep position. This allowed us to validate that positional therapy applied with a smartphone helped reducing the supine position and improve the ODI severity. We revealed how a high-resolution sleep position is necessary to better assess the patient position and the occurrence of desaturation events. The novel results presented in this study suggest that smartphones are promising mHealth tools that can provide clinically relevant information by monitoring sleep position and its relationship with sleep disorders at home.

## 6. Patents

The Android application designed in this study and the algorithms used are under a process to recognize the industrial property.

## Figures and Tables

**Table 1:** Time spent in minutes (Min) and percentage (%), and sleep angle’s mean value ($\bar{X}$) and standard deviation ($\sigma_{X}$) for each sleep position and each patient. The time spent in minutes and percentage is also provided for the laying and standing conditions.

Subjects	Left				Supine				Right				Prone				Laying		Standing	
	Min	%	$\bar{X}$	$\sigma_{X}$	Min	%	$\bar{X}$	$\sigma_{X}$	Min	%	$\bar{X}$	$\sigma_{X}$	Min	%	$\bar{X}$	$\sigma_{X}$	Min	%	Min	%
1	16.0	4.3	56.2	1.1	210.5	56.7	94.6	9.2	142.4	38.4	157.1	4.3	2.3	0.6	−85.6	1.3	371.2	94.1	23.3	5.9
2	29.2	11.0	31.0	2.9	147.8	55.5	105.7	8.8	89.6	33.6	129.6	4.4	-	-	-	-	266.6	99.0	2.6	1.0
3	159.2	47.7	22.1	11.6	152.4	45.7	98.0	12.2	22.0	6.6	−173.1	16.1	-	-	-	-	333.6	100.0	-	0.0
4	-	-	-	-	114.5	36.1	112.4	1.6	202.6	63.9	161.4	6.2	-	-	-	-	317.1	100.0	-	0.0
5	87.3	30.7	59.2	1.2	145.4	51.1	85.7	18.2	51.9	18.2	157.9	2.4	-	-	-	-	284.6	100.0	-	0.0
6	59.4	19.1	5.6	5.0	90.6	29.1	97.4	4.2	46.9	15.1	175.5	20.7	114.4	36.8	−97.8	3.1	311.3	99.7	0.8	0.3
7	-	-	-	-	177.1	56.1	99.0	8.7	138.7	43.9	170.2	9.1	-	-	-	-	315.8	99.7	0.9	0.3
8	-	-	-	-	72.8	23.4	102.7	2.1	238.6	76.6	161.3	7.4	-	-	-	-	311.4	100.0	-	0.0
9	227.8	42.3	40.7	10.5	176.0	32.7	98.8	14.6	134.1	24.9	173.8	13.3	-	-	-	-	537.9	99.2	4.5	0.8
10	3.5	1.1	52.7	8.0	290.0	95.2	86.2	7.3	11.2	3.7	162.0	27.3	-	-	-	-	304.7	100.0	-	0.0
11	97.8	21.6	33.7	4.2	198.5	43.9	98.2	7.9	155.8	34.5	172.0	7.5	-	-	-	-	452.1	98.1	8.8	1.9
12	-	-	-	-	335.7	94.4	95.1	4.1	19.9	5.6	125.5	2.6	-	-	-	-	355.6	99.1	3.3	0.9
13	24.4	7.5	58.8	0.8	293.3	90.1	85.2	13.6	7.7	2.4	128.3	0.3	-	-	-	-	325.4	99.0	3.2	1.0
14	42.8	12.9	-22.1	14.5	29.0	8.7	86.5	5.0	185.6	55.8	174.2	22.2	75.4	22.7	−115.0	21.4	332.8	98.7	4.3	1.3
15	113.3	37.6	18.5	12.1	98.1	32.6	91.9	9.8	89.8	29.8	129.2	0.8	-	-	-	-	301.2	100.0	0.0	0.0
16	127.5	27.2	6.6	8.4	177.8	37.9	86.4	8.1	163.7	34.9	147.9	15.6	-	-	-	-	469.0	98.2	8.7	1.8
17	103.9	24.1	20.5	5.4	91.9	21.3	87.2	7.4	235.5	54.6	161.8	5.1	-	-	-	-	431.3	99.2	3.3	0.8
Total	1092.1	18.1	26.6	20.7	2801.4	46.5	94.0	12.2	1936.0	32.2	160.6	17.5	192.1	3.2	−104.4	16.1	6021.6	99.0	63.7	1.0

**Table 2:** Number of desaturation events, sleep time, ODI, and ODI severity for each of the subjects of the database.

Subjects	Desaturation Events (Number)	Sleep Time (Hours)	ODI (h^−1^)	ODI Severity
1	92	6.2	14.9	Mild
2	107	4.4	24.1	Moderate
3	43	5.6	7.7	Mild
4	136	5.3	25.7	Moderate
5	4	4.7	0.8	Healthy
6	6	5.2	1.2	Healthy
7	138	5.3	26.2	Moderate
8	108	5.2	20.8	Moderate
9	42	9.0	4.7	Healthy
10	20	5.1	3.9	Healthy
11	295	7.5	39.2	Severe
12	342	5.9	57.7	Severe
13	83	5.4	15.3	Moderate
14	40	5.5	7.2	Mild
15	78	5.0	15.5	Moderate
16	230	7.8	29.4	Moderate
17	203	7.2	28.2	Moderate
Total/Mean	1967	100.3	19.6	Moderate

**Table 3:** Time spent in each sleep position in minutes (Min) and percentage (%), number of desaturation events, total sleep time, ODI, and ODI severity for the subset of subjects who underwent positional treatment.

Subjects	Left		Supine		Right		Prone		Desaturation Events (Number)	Sleep Time (Hours)	ODI (h^−1^)	ODI Severity
	Min	%	Min	%	Min	%	Min	%				
1	165.9	34.6	29.7	6.2	283.9	59.2	-	-	74	8.0	9.3	Mild
2	264.4	55.3	3.6	0.8	210.4	44.0	-	-	246	8.0	30.9	Severe
3	183.9	40.4	19.9	4.4	251.4	55.2	-	-	89	7.6	11.7	Mild
4	156.9	45.6	4.0	1.2	177.7	51.6	5.5	1.6	72	5.7	12.6	Mild
5	73.9	16.1	6.1	1.3	204.6	44.6	173.7	37.9	17	7.6	2.2	Healthy
6	80.5	15.2	2.4	0.4	189.2	35.6	258.8	48.8	18	8.8	2.0	Healthy
7	185.4	44.5	0.4	0.1	230.7	55.4	-	-	48	6.9	6.9	Mild
10	59.7	19.7	9.8	3.2	143.3	47.7	89.6	29.6	36	5.0	7.1	Mild
13	195.1	59.5	0.8	0.2	121.0	36.9	10.9	3.3	91	5.5	16.7	Moderate
Total	1365.7	36.0	76.7	2.0	1812.2	47.8	538.5	14.2	691	63.2	10.9	Mild
